# Yellow fever virus resurgence in Sao Paulo State, Brazil, 2024–2025

**DOI:** 10.1590/S1678-9946202668013

**Published:** 2026-02-16

**Authors:** Mariana Sequetin Cunha, Juliana Mariotti Guerra, Márcio Junio Lima Siconelli, Benedito Antonio Lopes da Fonseca, Jessica Caroline de Almeida Dias, Gisele Dias de Freitas, Ester Cerdeira Sabino, Erika Regina Manuli, Geovana Maria Pereira, Ian Nunes Valença, Patrícia Sayuri Silvestre Matsumoto, Nuno Rodrigues Faria, Natália Coelho Couto de Azevedo Fernandes

**Affiliations:** 1 Instituto Adolfo Lutz, São Paulo, São Paulo, Brazil; 2 Universidade de São Paulo, Faculdade de Medicina de Ribeirão Preto, Ribeirão Preto, São Paulo, Brazil; 3 Centro de Vigilância Epidemiológica Prof. Alexandre Vranjac, São Paulo, São Paulo, Brazil; 4 Universidade de São Paulo, Faculdade de Medicina, Instituto de Medicina Tropical de São Paulo, São Paulo, São Paulo, Brazil; 5 Saint Mary's University, Department of Geography and Environmental Studies, Halifax, Canada; 6 Imperial College London, School of Public Health, Department of Infectious Disease Epidemiology, MRC Centre for Global Infectious Disease Analysis, London, UK

**Keywords:** Yellow fever virus, Phylogenetics, Surveillance

## Abstract

We report the detection of yellow fever virus in Campinas and Ribeirao Preto city areas (two geographic areas within Sao Paulo State, Brazil) from September 2024 to February 2025. Phylogenetic analysis of six new genomes showed a re-introduction in 2022 from Midwest Brazil followed by persistence in Sao Paulo State. Continued surveillance in neotropical primates is required to prevent cases in humans.

## INTRODUCTION

Yellow fever (YF) is a severe disease caused by *Orthoflavivirus flavi* (formerly known as the yellow fever virus, YFV), a member of the *Flaviviridae* family. It remains endemic in parts of Africa and the Americas, posing an ongoing public health threat^1^. In Brazil, the urban cycle of YF, transmitted by *Aedes aegypti* mosquitoes, was eradicated in 1942. Since then, YFV has persisted in a sylvatic cycle involving several neotropical primate species (NTPs) and forest canopy-dwelling mosquitoes, primarily *Haemagogus* spp. and *Sabethes* spp.^2^. Consequently, YF surveillance relies on the confirmation of epizootic events with virus detection via RT-qPCR and/or immunohistochemistry following the guidelines of the Brazilian Ministry of Health^3^. Despite the availability of the live attenuated 17DD vaccine, from mid-2016 up to late 2018, Brazil experienced one of the largest YF outbreaks in recent decades, predominantly affecting southeastern Brazil^4–7^. This outbreak was driven by the 1E lineage of the South American I genotype that originated from the Amazon basin and spread from northern Sao Paulo into neighboring areas of western Minas Gerais and southern Sao Paulo states, reaching areas without vaccine recommendations at the time^8^. More recently, in March 2023, a *Callicebus nigrifrons* primate from Aguas da Prata municipality (Sao Paulo State border with Minas Gerais State) tested positive for YFV. Full genome analysis indicated a new introduction from the Brazilian Midwest^9^. Since September 2024, YFV cases have been detected in humans and NTPs in two Sao Paulo State regions: Campinas surrounding area (Car) and Ribeirao Preto surrounding area (RPar). This study describes phylogenetic findings from YFV genomes obtained in 2024-2025, indicating the persistence of a new lineage that was likely introduced from the Brazilian Midwest. Additionally, the growing number of cases in human and nonhuman primates confirms the expanding boundaries of YF circulation, highlighting continued spillover risk in locations near large urban centers.

### Ethics

Research on residual anonymized human samples collected at Hospital das Clinicas and sequenced at Instituto de Medicina Tropical de Sao Paulo (IMTSP), Universidade de Sao Paulo, was approved by the HC-FMUSP Ethics in Research Committee (N° 2.669.963, 3.258.615, and 3.371.745).

## MATERIALS AND METHODS

**Figure 1:**
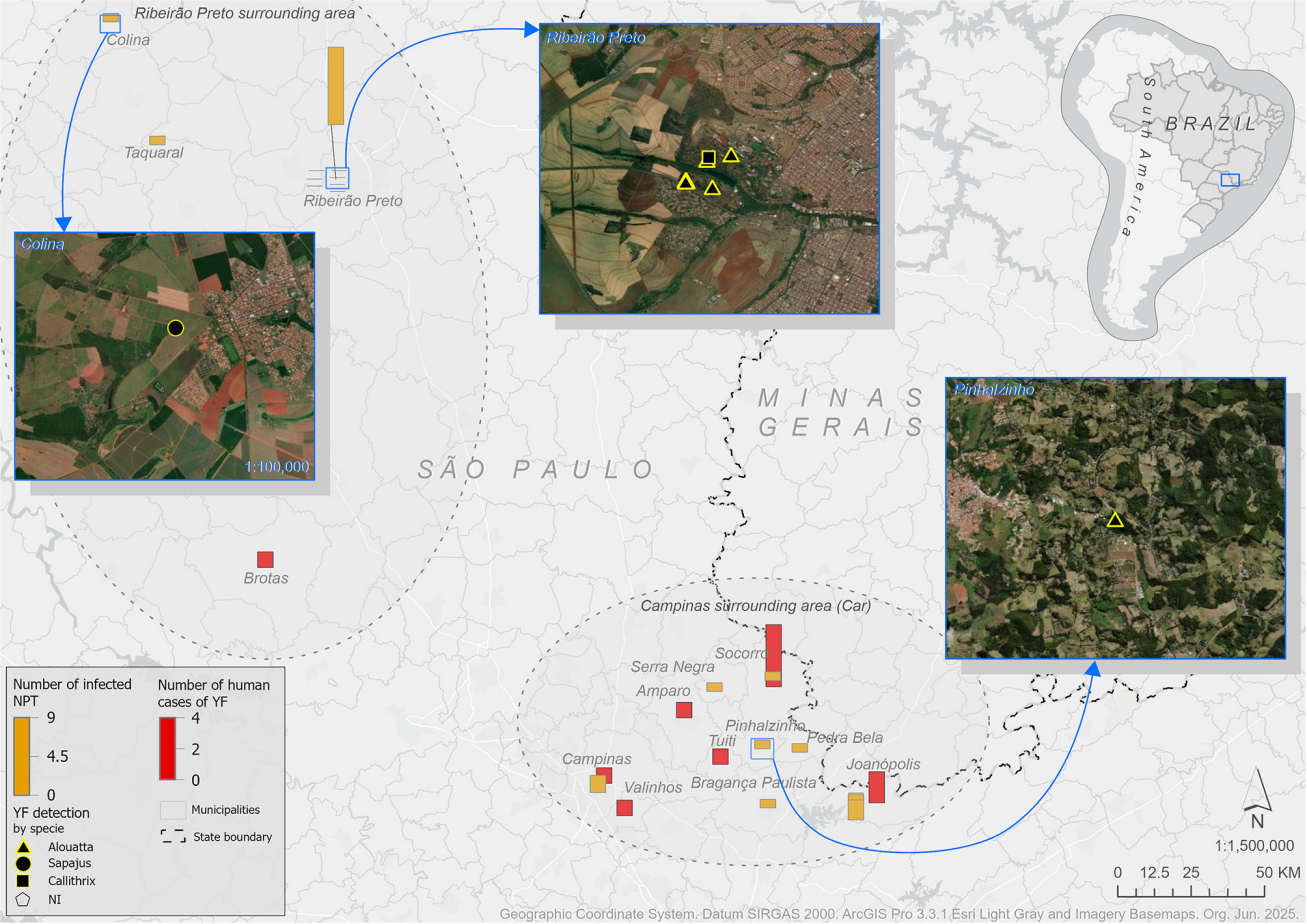
Map of Sao Paulo State showing the Campinas (Car) and Ribeirao Preto surrounding areas (RPar). The map was created using ArcGIS Pro 3.3.1. ESRI Basemaps as sources 297 × 209mm (300 × 300 DPI)

On September 18 and 24, 2024, liver samples of two nonhuman primates collected in the Sao Paulo State municipalities Pedra Bela (Car) and Braganca Paulista (Car), respectively, tested positive for YFV by RT-qPCR^10^ and/or immunohistochemistry. Liver samples were processed by Instituto Adolfo Lutz, as previously described^4^. In late December 2024, epizootic events (all *Alouatta caraya* primates) were confirmed in Ribeirao Preto (RPar), 200 Km away from the cases detected three months earlier. As of 13 February 2025, 21 nonhuman primates tested positive for YFV RNA (*Alouatta* = 12, 57.1%; *Callithrix* = 5, 23.8%; *Sapajus* = 3; 14.3%; 2 not informed), and YF activity has been detected in Pinhalzinho (Car), Socorro (Car), Campinas (Car), Colina (RPar), Ribeirao Preto (RPar), Serra Negra (Car), and Joanopolis (Car), whereas 11 human cases were detected, with eight deaths, all in Car (Figure 1).

For whole genome sequencing, cDNA synthesis, library preparation for six cases was performed at the Laboratorio Estrategico at Instituto Adolfo Lutz (Supplementary Table S1). YFV genomes were generated using hybridization-based library preparation and capture sequencing with the Viral Surveillance Panel v.2 (Illumina, San Diego, CA, USA). Consensus sequences were constructed on Geneious Prime v.2024.0.2 using the YFV 17D vaccine strain reference sequence NC_002031.1. One additional human sequence from January 2025—generated with viral metagenomics^11^—was also included. Consensus sequences were aligned using MAFFT v.7^12^. Maximum likelihood phylogenetic analyses were conducted using IQ-TREE2 v.2.2.2.6^13^. Dating phylogenetic analyses were performed using BEAST 1.10.4^14^ with the BEAGLE 3 library for computational efficiency^15^. We used an HKY nucleotide substitution model with gamma-distributed rate heterogeneity and applied an uncorrelated relaxed molecular clock and a skygrid coalescent tree model prior, incorporating the CTMC scale reference prior^16^. Sequences were deposited on NCBI GenBank under accession N° PQ602526, PQ963937, PQ879115-PQ879118. Ethical committee approval for animal diagnosis was not required for this study as the detection of YFV in non-human primates was performed exclusively using samples from routine public health surveillance.

**Figure 2:**
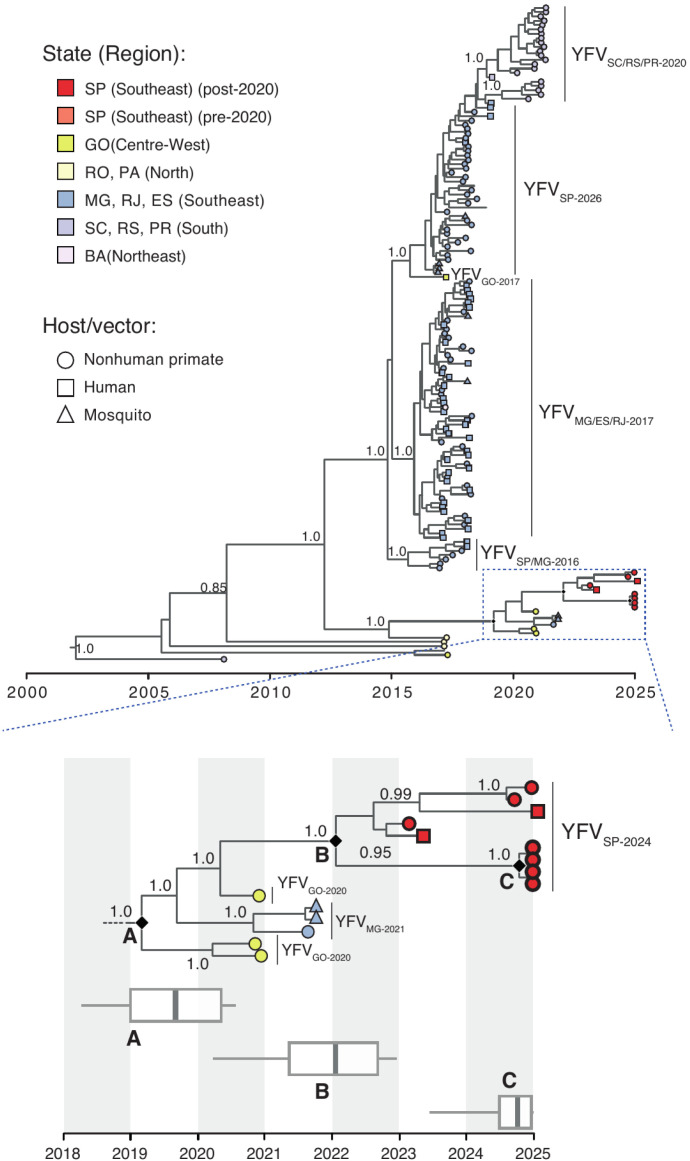
Time-scaled Bayesian phylogeny of YFV sequences from Brazil. Tip colors correspond to Brazilian states/regions, as defined in the key. Tip symbols indicate host/vector type (circle = nonhuman primate, square = human, triangle = mosquito). Clades are defined as previously (28), now including year of first detection for each clade. Posterior support values are shown at key nodes. Inset below shows a zoomed view of clades A–C (note that Clade C is nested within B and B is nested within A). Boxes along the timeline indicate 95% Bayesian credible intervals for the time to the most recent common ancestor of each clade. See Supplementary Table S1 for geographic coordinates and host information for new sequences. Mean evolutionary rate was estimated at 6.62 × 10^-4^ substitutions per site per year (95% Bayesian credible interval: 5.24 × 10^-4^ to 8.08 × 10^-4^).

Phylogenetic analysis showed that the 2024-2025 Sao Paulo sequences all clustered with maximum statistical support (posterior probability = 1.0) in a monophyletic cluster (Clade B) (Figure 2, Supplementary Figure S1). This clade is most closely related to a sequence from Goias State (Midwest Brazil) that is genetically distinct from the viruses circulating during the 2016–2018 epidemic in Sao Paulo State. The estimated date of the most recent common ancestor (TMRCA) of Clade B was around January 2022 (95% Bayesian credible interval, BCI, April 2021 to Aug. 2022). The recent 2024-2025 YFV genomes from RPar (Clade C) clustered within Clade B. This suggests that following a reintroduction from the Midwest around early 2022, YFV has persisted and continued to spread within the Sao Paulo State. Our analyses also show that Clade B has split off from a more basal Clade A around August 2019 (95% BCI December 2018 to March 2020), which also contains additional sequences from Goias (coordinates −16.041192; −49.622508) and Minas Gerais (coordinates −18.456178; −44.673361) states (Figure 2).

## DISCUSSION

Our phylogenetic analysis confirms the sustained circulation of a distinct YFV lineage (Clade B) in Sao Paulo State since its reintroduction from the Brazilian Midwest in or around early 2022. This lineage has diverged from sequences obtained from Goias and Minas Gerais states (Clade A) and now dominates transmission in the Campinas (Car) and Ribeirao Preto (RPar) regions. The monophyletic clustering of 2024–2025 sequences (Clade C within Clade B) suggests a recent continuous local evolution within Sao Paulo State that is independent from the lineages circulating during the 2016–2018 epidemic^17^. This persistence may reflect pockets of susceptible nonhuman primate populations and/or adaptive changes facilitating viral maintenance across ecological niches, particularly in fragmented forest edges and riparian forest areas where competent *Haemagogus* vectors and nonhuman primate hosts overlap.

Our findings have significant public health implications. The detection of YFV in 21 nonhuman primates and 11 humans across seven municipalities in Sao Paulo State (Figure 1) shows continued spatial spread beyond historical risk zones. Critically, human deaths were in non-vaccinated individuals^18^, underscoring gaps in immunization coverage despite vaccine availability. The YFV incursion into densely populated peri-urban areas like Campinas increases the risks of urban spillover via *Aedes aegypti*.

Our findings reinforce the need to boost surveillance strategies to democratize genomic surveillance, including the rapid sequencing of human, vector, and nonhuman primate samples to better understand the pathways of lineage spread across the region. The surveillance of sentinel NTPs remains the gold standard in early warning systems for detecting YFV circulation. However, it relies on the field and laboratory capacity of local authorities and the prompt response of reference laboratories. The Midwest-to-Sao Paulo reintroduction dispersal route (evinced by the putative origin of Clade B in Goias State) suggests ongoing viral traffic along ecological corridors^19^ and requires regional and inter-regional coordination. Targeted campaigns in expanding epizootic zones prioritizing forest-adjacent communities and travelers are essential, as implemented in 2017 in Sao Paulo state parks. Finally, due to the complex transmission cycle of YFV, its surveillance requires a One Health approach.

The predominance of *Alouatta* spp. deaths (57% of the nonhuman primate cases detected in our study) further highlights their role as amplification hosts as in Brazil YFV fatality rate in *Alouatta* genus is very high^5^. However, finding YFV in *Sapajus* and *Callithrix*, two genera in peri and urban sites that may increase the risk of YFV re-urbanization, is alarming. Epizootic surveillance based on the early detection and notification of YFV in NTPs should be encouraged and strengthened across all regions. Real-time mortality reporting of nonhuman primates by citizen science using SISS-Geo^20^ could further support and trigger pre-emptive human vaccination campaigns.

## CONCLUSION

Our findings show a resurgence of YFV activity in previously unaffected municipalities of Sao Paulo State, underscoring its continued presence in Southeast Brazil since its reintroduction in 2022. This ongoing circulation highlights the urgent need for coordinated surveillance that integrates data from nonhuman primates, vectors, and human populations. Strengthening vaccination campaigns and enhancing monitoring across Midwest and Southeast transmission corridors are essential to reduce the risk of further human fatalities and to prevent the possible re-establishment of urban transmission.

## Data Availability

The complete anonymized dataset supporting the findings of this study is available from https://doi.org/10.48331/SCIELODATA.KDQJTE
