# Supplementary material for: Yellow fever virus resurgence in Sao Paulo State, Brazil, 2024–2025
Source: Rev Inst Med Trop Sao Paulo. 2026 Feb 16;68:e13. doi: 10.1590/S1678-9946202668013 (PMC12919302; doi:10.1590/S1678-9946202668013)
Supplement: Supplementary Material 1 [file 1678-9946-rimtsp-68-S1678-9946202668013-Suppl01.pdf]

REVISTA DO INSTITUTO DE MEDICINA TROPICAL DE SÃO PAULO
JOURNAL OF THE SÃO PAULO INSTITUTE OF TROPICAL MEDICINE

SUPPLEMENTARY MATERIAL

https://doi.org/10.48331/SCIELODATA.KDQJTE

Yellow fever virus resurgence in Sao Paulo State, Brazil, 2024–2025

Mariana Sequetin Cunha[1], **Juliana Mariotti Guerra**[1], **Márcio Junio Lima Siconelli**[2], **Benedito Antonio Lopes da Fonseca**[2], **Jessica Caroline de Almeida Dias**[2], **Gisele Dias de Freitas**[3], **Ester Cerdeira Sabino**[4], **Erika Regina Manuli**[4], **Geovana Maria Pereira**[4], **Ian Nunes Valença**[4], **Patrícia Sayuri Silvestre Matsumoto**[5], **Nuno Rodrigues Faria**[4,6], **Natália Coelho Couto de Azevedo Fernandes**[1]

[1]Instituto Adolfo Lutz, São Paulo, São Paulo, Brazil

[2]Universidade de São Paulo, Faculdade de Medicina de Ribeirão Preto, Ribeirão Preto, São Paulo, Brazil

[3]Centro de Vigilância Epidemiológica Prof. Alexandre Vranjac, São Paulo, São Paulo, Brazil

[4]Universidade de São Paulo, Faculdade de Medicina, Instituto de Medicina Tropical de São Paulo, São Paulo, São Paulo, Brazil

[5]Saint Mary's University, Department of Geography and Environmental Studies, Halifax, Canada

[6]Imperial College London, School of Public Health, Department of Infectious Disease Epidemiology, MRC Centre for Global Infectious Disease Analysis, London, UK

Correspondence to: Mariana Sequetin Cunha
Instituto Adolfo Lutz, Av. Dr. Arnaldo, 355, CEP 01246-000, São Paulo, SP, Brazil
Tel: +55 11 3068-2993
E-mail: masequetin@gmail.com

Received: 22 May 2025
Accepted: 3 December 2025

Editor: Camila Malta Romano

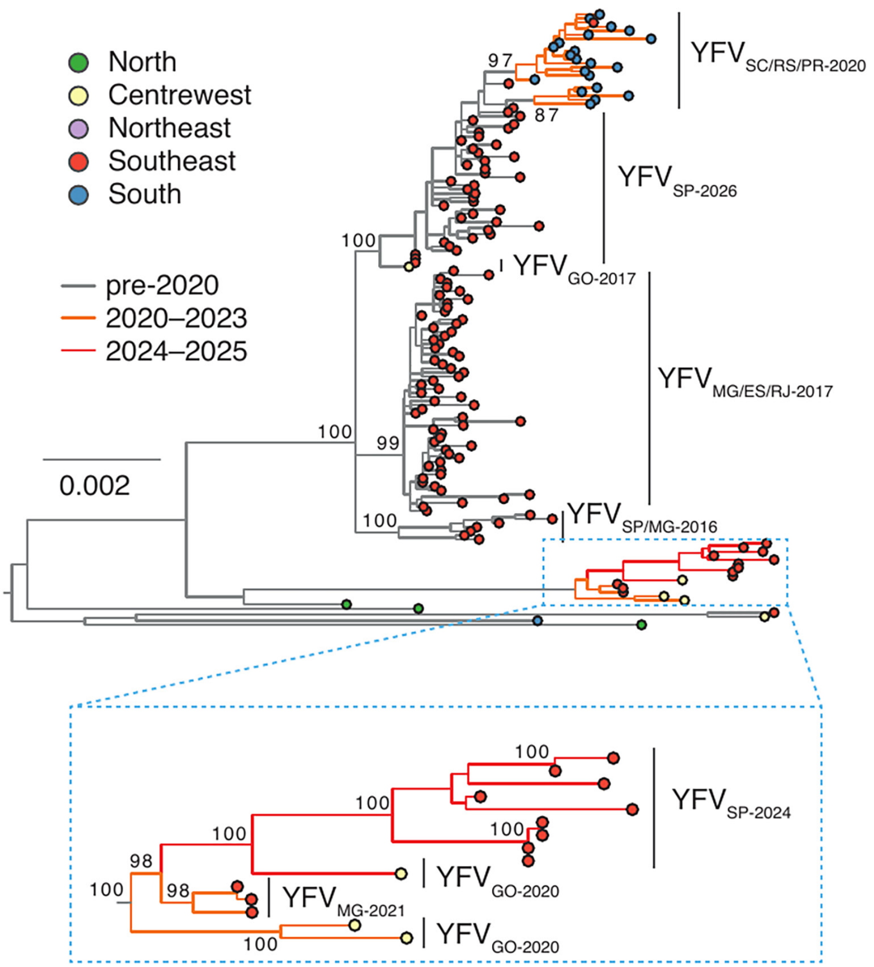

Supplementary Figure S1 - Maximum likelihood phylogeny of YFV genomes from Brazil. Phylogenetic tree inferred from 151 full-length YFV genomes, showing major spatiotemporal lineages from 2016–2025 (denoted in Figure 2). Tip colors represent the geographic region of sampling (North, Midwest, Northeast, Southeast, South). Branch colors indicate sampling period: grey (pre-2020), orange (2020–2023), and red (2024–2025). Support values at key nodes indicate ultrafast bootstrap (UFBoot) percentages. The inset at the bottom shows an expanded view of the most recent strains and the clear separation of the SP-2024 sublineage. The scale bar represents substitutions per site.

This is an open-access article distributed under the terms of the Creative Commons Attribution License.

Supplementary Table S1 – Accession numbers and metadata of sequences in this study, 2024

Accession number	Host	Location	Coordinates	Area	Date of sampling
PQ602526	*Alouatta* sp.	Pedra Bela	–22.775758; –46.441848*	Car	18 Sep 2024
PQ963937	*Alouatta* sp.	Pinhalzinho	–22.78642; –46.559196	Car	20 Dec 2024
PQ879115	*Alouatta caraya*	Ribeirao Preto	–21.16855; –47.86516	RPar	26 Dec 2024
PQ879116	*Alouatta caraya*	Ribeirao Preto	–21.16855; –47.86516	RPar	26 Dec 2024
PQ879117	*Alouatta caraya*	Ribeirao Preto	–21.16855; –47.86516	RPar	26 Dec 2024
PQ879118	*Alouatta caraya*	Ribeirao Preto	–21.16855; –47.86516	RPar	26 Dec 2024

*Coordinates from the municipality centroid.
